# Antioxidant and Antidiabetic Potential of the Antarctic Lichen *Gondwania regalis* Ethanolic Extract: Metabolomic Profile and In Vitro and In Silico Evaluation

**DOI:** 10.3390/antiox14030298

**Published:** 2025-02-28

**Authors:** Alfredo Torres-Benítez, José Erick Ortega-Valencia, Nicolás Jara-Pinuer, Jaqueline Stephanie Ley-Martínez, Salvador Herrera Velarde, Iris Pereira, Marta Sánchez, María Pilar Gómez-Serranillos, Ferdinando Carlo Sasso, Mario Simirgiotis, Alfredo Caturano

**Affiliations:** 1 Carrera de Química y Farmacia, Facultad de Ciencias, Universidad San Sebastián, General Lagos 1163, Valdivia 5090000, Chile; 2 Tecnológico Nacional de México, Instituto Tecnológico Superior de Xalapa, Sección 5ª Reserva Territorial S/N Col. Santa Bárbara, Xalapa-Enríquez 91096, Veracruz, Mexico; erick.ortega@itsx.edu.mx (J.E.O.-V.); stephanie.ley@itsx.edu.mx (J.S.L.-M.); salvador.hv@xalapa.tecnm.mx (S.H.V.); 3 Instituto de Farmacia, Facultad de Ciencias, Universidad Austral de Chile, Campus Isla Teja, Valdivia 5090000, Chile; nickbleed@gmail.com (N.J.-P.); mario.simirgiotis@uach.cl (M.S.); 4 Instituto de Ciencias Biológicas, Universidad de Talca, Av. Lircay s/n, Talca 3460000, Chile; ipereira@utalca.cl; 5 Departamento de Farmacología, Farmacognosia y Botánica, Facultad de Farmacia, Universidad Complutense de Madrid, Plaza Ramón y Cajal s/n, Ciudad Universitaria, 28040 Madrid, Spain; pserra@ucm.es (M.S.); martas15@ucm.es (M.P.G.-S.); 6 Department of Advanced Medical and Surgical Sciences, University of Campania Luigi Vanvitelli, 80138 Naples, Italy; ferdinando.sasso@unicampania.it; 7 Department of Human Sciences and Promotion of the Quality of Life, San Raffaele Roma Open University, 00166 Rome, Italy

**Keywords:** *Gondwania regalis*, ethanolic extraction, metabolomic profiling, antioxidant capacity, antidiabetic activity, in vitro assays, molecular docking and dynamics

## Abstract

Lichens are an important source of diverse and unique secondary metabolites with recognized biological activities through experimental and computational procedures. The objective of this study is to investigate the metabolomic profile of the ethanolic extract of the Antarctic lichen *Gondwania regalis* and evaluate its antioxidant and antidiabetic activities with in vitro, in silico, and molecular dynamics simulations. Twenty-one compounds were tentatively identified for the first time using UHPLC/ESI/QToF/MS in negative mode. For antioxidant activity, the DPPH assay showed an IC_50_ value of 2246.149 µg/mL; the total phenolic content was 31.9 mg GAE/g, the ORAC assay was 13.463 µmol Trolox/g, and the FRAP assay revealed 6.802 µmol Trolox/g. Regarding antidiabetic activity, enzyme inhibition yielded IC_50_ values of 326.4513 µg/mL for pancreatic lipase, 19.49 µg/mL for α-glucosidase, and 585.216 µg/mL for α-amylase. Molecular docking identified sekikaic acid as the most promising compound, with strong binding affinities to catalytic sites, while molecular dynamics confirmed its stability and interactions. Toxicological and pharmacokinetic analyses supported its drug-like potential without significant risks. These findings suggest that the ethanolic extract of *Gondwania regalis* is a promising source of bioactive compounds for developing natural antioxidant and antidiabetic therapies.

## 1. Introduction

Lichens are symbiotic organisms composed of a fungus (mycobiont), an alga (phycobiont), and/or cyanobacteria (cyanobiont), along with a microbiome that influences their adaptation, growth, and the presence of secondary metabolites, most of which are specific [1,2,3,4]. They exhibit high taxonomic and phylogenetic diversity, which is classified within the phyla Basidiomycota and Ascomycota [5]. In the Antarctic continent, approximately 400 species have been reported, from studies in the 1980s [6] to more recent investigations post-2000 [7,8], with the highest number of reports from King George Island in the South Shetland Islands archipelago. Additionally, according to biogeographic studies, there are species with common ecological similarity and distribution patterns shared between Patagonia and the Maritime Antarctic regions [9].

In general, lichens, due to their complex chemical composition mainly consisting of aromatic compounds such as depsides, tridepsides, depsidones, dibenzofurans, terpenes, and steroids, derived from specific metabolic pathways [10,11,12], represent promising sources of bioactive compounds with various biological activities [13]. These compounds possess properties, especially antioxidant, antimicrobial, anticancer, antidiabetic, and neuroprotective effects [14,15,16]. The impact of the ethnopharmacological approach to lichens has gained relevance in recent years, with reports of their use in human and animal nutrition, industrial applications, and as bioindicators of air quality, among others [17,18,19,20]. Additionally, there is evidence of the high biological potential of major compounds present in lichen extracts, such as atranorin and the acids barbatic, diffractaic, evernic, fumarprotocetraric, lobaric, usnic, and vulpinic. These compounds have been chemically characterized and scaled up in in vitro, in vivo, and in silico studies [21].

Complementarily, metabolomic studies and the combination of advanced chromatographic and mass spectrometry techniques [22] have identified the chemical variability of lichens and its relationship with their biological properties, highlighting their importance in the bioprospecting of new bioactive molecules with pharmacological effects on diseases with high prevalence and incidence, such as those related to the central nervous system, metabolic syndrome, and cancer, among others [23,24,25].

It is evident that the chemical and biological exploration of lichens is highly variable, considering their habitat and morphology (biotype), where factors such as nutrient availability, solar radiation, and humidity directly influence the production of secondary metabolites [26]. *Gondwania regalis*, along with four other species of the family Telochistaceae, belong to the group of saxicolous lichens that colonize rocks and can adapt and survive in extreme conditions [27]. Among the most studied species in similar habitats are *Umbilicaria antarctica*, *Ochrolechia frigida*, *Rhizocarpon geographicum*, and *Placopsis contortuplicata*, known for their richness in compounds with high biological potential [28,29,30].

This study aimed to evaluate the metabolomic profile of the ethanolic extract of the Antarctic lichen *Gondwania regalis* and assess its antioxidant and antidiabetic potential through in vitro assays, complemented by molecular docking, and molecular dynamics simulations. The novelty of this work lies in the first contribution to the phytochemical profile of *G. regalis* and its potential effect on two pharmacological activities of great interest to health.

## 2. Materials and Methods

### 2.1. Lichen Material

The species was collected on Ardley Island, Maxwell Bay, located on King George Island in the South Shetland Islands archipelago on rocks (62°12′35.6″ S; 58°55′58.4″ W) at 9 masl. Leg. A. Torres, Det. I. Pereira, vouchers no. 1158, 1159, Herbarium UTALCA. The lichen material was cleaned using dissecting forceps to remove any remaining material adhering to the thallus and a brush to remove any adhering dust. It was then dried in the shade in an airy place, stored in paper bags. and kept at room temperature.

### 2.2. Taxonomic Determination

The sample collected in the field was taken to the laboratory and identified, analyzing morphological, anatomical, ecological characters, reproductive strategies, determination of the photobiont, and colorimetric tests. For morphological examination of reproductive structures such as the apothecia and pycnidia, they were cut by hand and observed in water, as well as paraphysis tips and cortical tissues. Measurements were made for ascospores, pycnidiospores, and paraphyses, and for larger structures such as the thecium height and exciple width. The color of the epithecium and hypothecium were also observed. The total number of ascospores and pycnidiospores measured was 10, and the measurements given corresponded to the average of these. All the observations were made using a binocular optical microscope MOTIC model BA210, equipped with a graduated eyepiece and microphotographic camera. In addition, colorimetric tests were performed on the upper cortex and medulla of the thallus (K, C), and for this, we used a binocular magnifying glass brand L&T OPTIS. For the identification of this taxon, classic reviews, monographs, and dichotomous keys were used, and the taxonomic nomenclature follows the Index Fungorum.

### 2.3. Preparation of Ethanolic Extracts

A total of 5 g of *G. regalis* were macerated using ethanol as a solvent in an ultrasonic bath (80 kHz) at 40 °C, with three successive extractions of 50 mL, each lasting 30 min. The resulting ethanolic extracts were filtered, and the solvent was evaporated under reduced pressure at 38 °C, yielding a concentrated gummy extract.

### 2.4. LC Parameters and MS Parameters

The separation and identification of the compounds present in the ethanolic extracts were performed using a UHPLC-ESI-QToF-MS system. This system consisted of a UHPLC Ultimate 3000 RS with Chromeleon 6.8 software (Dionex GmbH, Idstein, Germany) coupled to a Bruker maXis ESI-QToF-MS with Data Analysis 4.0 software (Bruker Daltonik GmbH, Bremen, Germany). A total of 5 mg of each extract was dissolved in 2 mL of analytical-grade methanol and filtered using polytetrafluoroethylene (PTFE) filters. Subsequently, 10 μL of this solution was injected into the system. The chromatographic system included a quaternary pump, an autosampler, a thermostatted column compartment, and a photodiode array detector. Elution was performed using a binary gradient system with mobile phase (A) 0.1% formic acid in water and mobile phase (B) 0.1% formic acid in acetonitrile, with the following gradient program: 1% B isocratic (0–2 min), 1–5% B (2–3 min), 5% B isocratic (3–5 min), 5–10% B (5–8 min), 10–30% B (8–30 min), 30–95% B (30–38 min), and 1% B isocratic (39–50 min). Separation was carried out using a Thermo 5 µm C18 80 Å column (150 mm × 4.6 mm) at a flow rate of 1.0 mL/min. ESI-QToF-MS experiments were conducted in negative ion mode, with a scan range from 100 to 1200 *m*/*z*. The electrospray ionization (ESI) conditions included a capillary temperature of 200 °C, a capillary voltage of 2.0 kV, a dry gas flow rate of 8 L/min, and a nebulizer pressure of 2 bar. The experiments were performed in automatic MS/MS mode. The structural characterization of phytocompounds was based on HR full MS, fragmentation patterns, and comparisons with the literature data.

### 2.5. Total Phenolic Content

The Folin–Ciocalteu assay was used to determine the total phenolic content in the samples. The oxidation of polyphenols resulted in a bluish coloration, which was quantified by spectrophotometry at 765 nm using a gallic acid standard curve. Results are expressed in mg GAE/g of lichen [31].

### 2.6. Antioxidant Activity

#### 2.6.1. Ferric-Reducing Antioxidant Power (FRAP) Assay

The assay was based on the reduction of the ferric 2,4,6-tripyridyl-s-triazine complex (Fe^3+^-TPTZ to Fe^2+^-TPTZ), which generates a blue coloration in the samples and was measured by spectrophotometry at 593 nm using a Trolox standard curve. Results are expressed in µmol Trolox/g of lichen [32].

#### 2.6.2. Oxygen Radical Absorbance Capacity (ORAC) Assay

This assay, which evaluates the peroxyl radical scavenging capacity, used 2,2′-Azo-bis (2-amidinopropane) dihydrochloride (AAPH) in the samples. Excitation and emission wavelengths were measured at 485 and 530 nm, respectively, using Trolox for the calibration curve. Results are expressed in µmol Trolox/g of lichen [33].

#### 2.6.3. DPPH Scavenging Activity

The 2,2-diphenyl-1-picrylhydrazyl (DPPH) radical was used, which loses its color as antioxidants donate protons. The reaction was measured by spectrophotometry at 515 nm using a gallic acid standard curve. Results are expressed in µg/mL, denoting the median inhibitory concentration (IC_50_) [34].

### 2.7. Enzymatic Inhibitory Activity

#### 2.7.1. α-Glucosidase Inhibition Assay

A stock solution of 20 U/mL of the α-glucosidase enzyme was prepared in 2 mL of buffer for subsequent dilution. Solutions were measured by spectrophotometry at 415 nm over a one-minute interval for a total of 20 min, using an acarbose standard curve. Results are expressed in µg/mL (IC_50_) [35].

#### 2.7.2. α-Amylase Inhibition Assay

The α-amylase enzyme was prepared at a concentration of 0.5 mg/mL by weighing 2.5 mg and dissolving it in 5 mL of 20 mM phosphate buffer solution at pH 6.9. Solutions were measured by spectrophotometry at 515 nm using an acarbose standard curve. Results are expressed in µg/mL (IC_50_) [36].

#### 2.7.3. Lipase Pancreatic Inhibition Assay

A pancreatic lipase enzyme solution was prepared at a concentration of 10 mg/mL in Tris-HCl buffer. Solutions were measured by spectrophotometry at 410 nm using an orlistat standard curve. Results are expressed in µg/mL (IC_50_) [37].

### 2.8. Calculation of the Pharmacological Properties and Risk Toxicity

The *G. regalis* phytochemicals identified by UHPLC/MS were evaluated and verified for their drug-likeness and pharmacological properties using the Osiris Data Warrior (version 5.5.0) computational tool and the PubChem databases (https://pubchem.ncbi.nlm.nih.gov/ (accessed on 27 September 2024)). All identified compounds were evaluated according to Lipinski’s rule of five, which allows us to establish that an orally active drug must meet the following criteria: the molecular weight of the drug candidate must be <500 Da, the cLogP must be <5, the number of hydrogen donor bonds should be <5, the number of hydrogen acceptor bonds should be <10, and the number of rotatable bonds should be <10 [38]. The identified phytochemicals were also subjected to in silico toxicological analysis using the Osiris Data Warrior (v 5.5.0) computational tool. The risks used in the toxicological analysis were mutagenicity, tumorigenicity, irritability, and reproductive effect. Those phytochemicals that presented a maximum violation of the criteria of Lipinski’s rule and that did not present any toxicological risk were considered as possible candidate inhibitors of α-amylase, α-glucosidase, and human pancreatic lipase and were analyzed by in silico analysis [39,40].

### 2.9. In Silico Analysis

For the molecular docking analysis, the two-dimensional structures of the phytochemicals that did not present more than one violation of Lipinsky’s rule and no risk of toxicity were prepared (3,5-dietoxybenzoic acid, alpha licanic acid, olivetolic acid, pinellic acid, porrigenic acid, and sekikaic acid) using ChemDraw 8.0 software (PerkinElmer Informatics, Waltham, MA, USA). The chemical structures of the ligands were saved in .mol format. They were imported into the Avogadro program (https://avogadro.cc, accessed on 10 June 2024) to optimize the molecular geometry of the ligands using the force field function MMFF94 [40,41]. All ligands after optimization were saved in .mol2 format, which was used for molecular docking analysis to inhibit the α-amylase, α-glucosidase, and human pancreatic lipase enzyme. The compound acarbose was used as reference inhibitors for the enzymes α-amylase and α-glucosidase, and the compounds methoxy undecyl phosphonic acid (MUP) and orlistat were as reference inhibitors for the enzyme human pancreatic lipase [40].

The crystal structures of α-amylase (PDB:2QV4), α-glucosidase (maltase) (PDB: 2QMJ), and human pancreatic lipase (PDB:1LPB) were obtained from the PDB database (http://www.rcsb.org/pdb (accessed on 27 September 2024)). These PDBs came cocrystallized with the ligands acarbose (reference inhibitor for α-amylase and α-glucosidase), MUP, and orlistat (reference inhibitors for human pancreatic lipase) [39,40]. This allowed us to perform targeted docking at the inhibition site of the reference inhibitors, allowing us to have information on the amino acids involved in inhibiting each of the enzymes.

Crystallized enzymes were optimized using UCSF Chimera software (v1.16, San Francisco, CA, USA); water molecules and active site ligands were removed from the crystallographic enzymes. Polar hydrogen atoms were added, considering the appropriate ionization states for basic and acidic amino acid residues [39,40].

After the preparation of the ligands and the crystallized enzymes, the molecular docking analysis was carried out. The analysis was carried out using the rigid crystalline enzyme structures and the flexible ligands where the torsion angles were identified and obtaining the 10 lowest energy conformations for each of the ligands. Each of the molecular docking with the enzymes were carried out separately and in triplicate, obtaining the best conformation and binding affinity (kcal/mol) for each of the ligands to be evaluated. The grid parameters were determined using as reference the inhibitors crystallized in each of the enzymes (acarbose for α-amylase and α-glucosidase; MUP and orlistat for human pancreatic lipase). The analysis and evaluation of the molecular docking results were carried out using the Biovia Discovery Studio (v20.1.0.19295, San Diego, CA, USA: Dassault Systemes, 2020) and Chimera X programs [40].

### 2.10. Molecular Dynamic Simulations

After molecular docking, we performed atomistic molecular dynamics simulations for each system using the CHARMM36m enzyme force field obtained from the MacKerell lab website (updated July 2022), in conjunction with the GROMACS software package version 2023.4; the topology and parameters of the ligand were prepared/obtained using the CGenFF web app (academic version). As a starting structure of each MD system, we used the most favorable binding/energetic configuration provided/obtained from the molecular docking process. Each MD run was performed using the enzyme (α-amylase, α-glucosidase, and human pancreatic lipase) and ligand (sekikaic acid) centered in a dodecahedral box. Structures were then solvated with the TIP3P water model with a minimum of 1.2 nm buffer solvation layer beyond the solute; each system was electrically neutralized, and no excess salt was added.

Each system was optimized using the standard steepest descent energy minimization, followed by a thermalization process during 5 ns using the NVT canonical ensemble. After reaching the desired equilibrium temperature, a further pre-equilibrated run for 5 ns in the NPT isobaric–isothermal ensemble was performed to reach the equilibrium hydrostatic pressure. During both equilibration stages, we imposed a restriction of fixing the position of heavy atoms to equilibrate the solvent and ions around the protein and ligand molecules. Finally, each system underwent a 30 ns simulation using the standard leap-frog integrator with integration step of 2 fs. Temperature was maintained at 310 K using the velocity-rescale thermostat and pressure at 1 bar with the Parrinello-Rahman barostat (2 ps relaxation time, 4.5 × 10^−5^ bar^−1^ compressibility). Periodic boundary conditions were applied in all spatial directions. The Lennard–Jones and Coulomb interactions were used and computed within a cutoff radius of 1.2 nm, and long-range electrostatic interactions were calculated using the particle mesh Ewald (PME) method. Conformational configurations were saved every 50 ps, giving a total of 600 configurations. Physical observables were obtained using standard GROMACS tools, and visualization analysis was performed with the VMD for LINUXAMD64, version 1.9.3.

### 2.11. Statistical Analysis

Each sample solution was measured in triplicate, and the results are reported as mean ± standard deviations (SD) using Microsoft Excel 2019 (Microsoft Office, Microsoft Corporation, Redmond, WA, USA). To compare the means, a one-way analysis of variance (ANOVA) was performed, followed by Tukey’s test at a significance level of *p* < 0.05, using GraphPad Prism 8 (GraphPad Software Corporation, La Jolla, CA, USA).

## 3. Results

### 3.1. Morphological, Ecological, and Taxonomic Description of G. regalis

#### 3.1.1. Morphological Description

**Figure 1:**
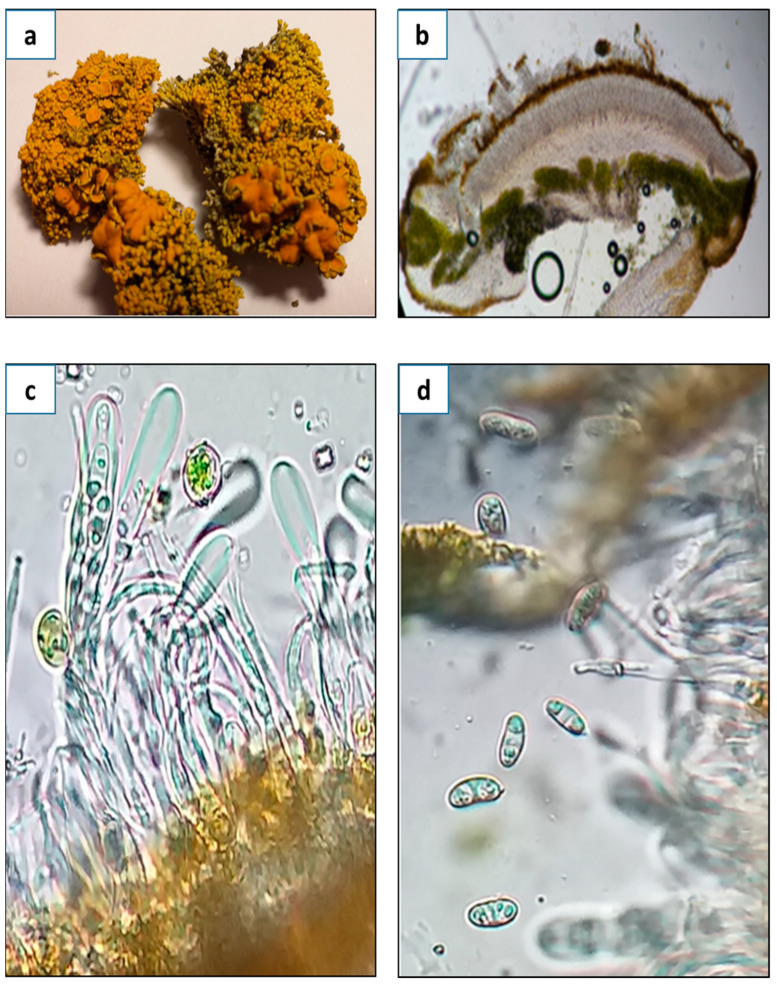
(**a**) Aspect of thallus and the apothecia of *G. regalis* (scale bar = 1 cm); (**b**) cross section of an apothecium of *G. regalis* (scale bar = 100 µm); (**c**) aspect of asci, paraphyses, and photobiont (scale bar = 10 µm); (**d**) polarilocular spores of *G. regalis* (scale bar = 10 µm).

The following taxonomic description is based, in part, on the lichen material that was used for the extraction and determination of primary and secondary metabolites. Thallus subfruticose: In the central part, dense vertical cushions were formed with a yellowish-orange upper surface, whitish lower surface and white medulla. The photobiont was green algae. An algal layer was formed by cells of 8–10 µm in diameter. Apothecia zeorine was numerous and very tightly located in the higher parts of the thallus, from 0.2, reaching between 0.4 and 0.6 mm in diameter, with margin thalline that was slightly crenate, more or less thick, of same color as the thallus, 200–250 µm; the disc was convex, then flat, and later on convex; it was brownish orange, and the epithecium was orange. Thecium hyaline was from 100 to 120 µm high, with hypothecium hyaline. Paraphyses was non-compact, thin, simple, and slightly bulging at the tips, 1–2 µm. Asci was cylindrical, sometimes slightly bulging at the apex or thinned; it was 80–90 µm long and 6–8 µm wide, with eight spores. Ascospores were polarilocular, hyaline, and narrowly ellipsoidal, at sizes of 10–12 × 4–6 µm. The septum was 5–6 µm. Picnidia was immersed in the lobe tips. Pycnidiospores were hyaline, ellipsoidal, at sizes of 2–2.5 × 1–1.5 µm. The spot test indicated thallus and apothecia K+ purple, C− (Figure 1).

#### 3.1.2. Ecology and Distribution

**Figure 2:**
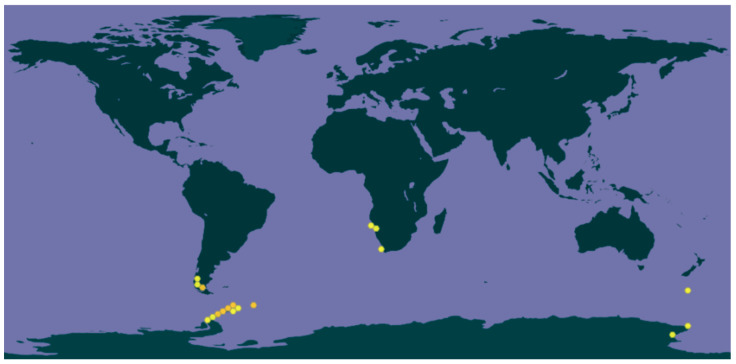
Distribution of *G. regalis* in the world (GBIF).

Saxicolous grows on eutrophicated rocks, even in cracks, along the coast where there is a strong presence of seabirds. It is characterized by forming thick, continuous yellowish-orange crusts. Its known distribution is from the Antarctic Peninsula, South Shetland Islands, and South Orkney Islands [42]. In Chile, this species is found in the region of Maga- llanes and marine Antarctica; between 30 and 300 m above sea level, it grows together with *Ramalina terebrata* and species of *Neuropogon*, province of Magallanes, Pali-Aike National Park. It is a species ornicotrophilous and can also appear along with *Xanthoria elegans* and *Haematomma erythromma*. There are also reports of the species in Namibia, South Africa, and New Zealand (Figure 2).

#### 3.1.3. Taxonomic Notes

*Gowdwania regalis* (Vain.) Søchting, Frödén, and Arup (=*Polycualiona regalis* (Vain.) Hue) was described and discussed by Poelt and Pelleter [43] as a species of *Caloplaca*. In 2013, it was transferred to the genus *Gondwania* by Arup et al. [44] based on molecular data (South American collections have been included in *G. regalis* by Poelt and Pelleter (1984), while Patagonian collections are described as a separate species, *Austroplaca imperialis*) [42].

### 3.2. Chromatographic Analysis of G. regalis

**Figure 3:**
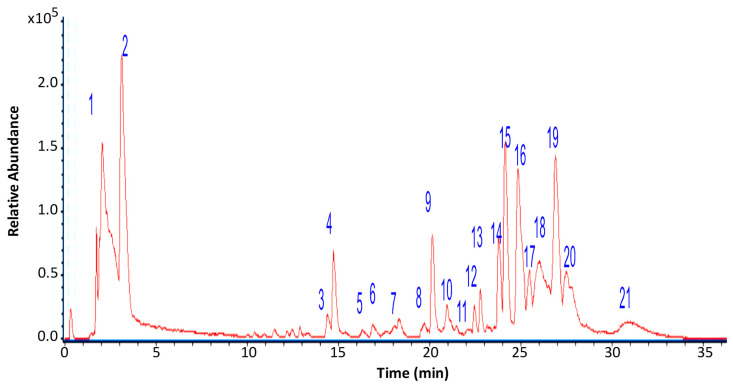
UHPLC/ESI/QToF/MS chromatogram of *G. regalis* ethanolic extract. The numbers above the peaks correspond to major compounds identified in the extract.

The chemical composition of the ethanolic extract of *G. regalis* (4% yield) was determined using high-resolution mass spectrometric analysis (UHPLC-MS) in negative mode (Figure 3). A total of 21 compounds were tentatively identified, classified as carbohydrates, organic acids, fatty acids, depsides, aromatics, iridoids, and dibenzofurans (Table 1).

**Carbohydrates:** Two carbohydrates were tentatively identified in the peaks 1 and 5 as mannitol and khellinol (C_6_H_13_O_6_ and C_13_H_9_O_5_), respectively.

**Organic Acid:** Two organics acids were tentatively identified as citric acid (C_6_H_7_O_7_, pick 2) and adipic acid (C_16_H_27_O_4_, pick 14).

**Fatty Acid:** Ten fatty acids were tentatively identified in the peaks 3, 6, 7, 8, 9, 16, 17, 19, 20, and 21 as azelaic acid, 9-octadecenedioic acid, pinellic acid, pinellic acid isomer, 2,4-dihydroxy-6-pentylbenzoate, 17-hydroxylinolenic acid, porrigenic acid, 18-hydroxylinoleic acid, 18-hydroxylinolenic acid, and alpha licanic acid (C_9_H_15_O_4_, C_18_H_31_O_4_, C_18_H_33_O_5_, C_18_H_33_O_5_, C_12_H_15_O_4_, C_18_H_29_O_3_, C_18_H_29_O_4_, C_18_H_3_1O_3_, C_18_H_29_O_3_, C_18_H_27_O_3_), respectively.

**Depside:** Two depsides were tentatively identified as lecanoric acid (C_16_H_13_O_7_, pick 11, and a molecular anion at *m*/*z* 317.0653) and sekikaic acid (C_22_H_25_O_8_, pixk 15, and a molecular anion at *m*/*z* 417.1571).

**Aromatic:** One aromatic was tentatively identified in the peak 12 as 3,5-dietoxybenzoic acid (C_11_H_13_O_4_).

**Iridoid:** One iridoid was tentatively identified as caryoptosidic acid (C_16_H_23_O_11_, pick 13, with a molecular anion at *m*/*z* 391.1245 and diagnostic peaks at *m*/*z* 311.2178, 263.1603).

**Dibenzofuran:** One dibenzofuran was tentatively identified as usnic acid (C_18_H_15_O_7_, peak 18, with a molecular anion at *m*/*z* 343.0822 and diagnostic peaks at *m*/*z* 295.2291, 231.0647 and 328.0570).

Additionally, two compounds were categorized as unknown.

### 3.3. Total Phenolic Content and Antioxidant Activity

Table 2 details the results of the antioxidant tests performed with the ethanolic extract of *G. regalis*. The phenolic content values indicate a moderately significant amount of phenolic compounds in the extract. Regarding FRAP and ORAC, the extract exhibited a moderate ability to donate electrons, reduce Fe^3+^ to Fe^2+^, and neutralize free radicals, respectively. In the DPPH assay, the extract was less efficient compared to gallic acid (IC_50_ of 2.24 ± 0.04).

### 3.4. Enzymatic Inhibitory Activity

Table 3 presents the enzymatic inhibition values obtained for the ethanolic extract of *G. regalis*. For α-glucosidase, a high inhibition was observed compared to acarbose as the reference compound (IC_50_ of 206.614 ± 0.008). In the case of α-amylase, the *G. regalis* extract showed lower inhibition compared to acarbose (IC_50_ of 6.477 ± 0.003), as well as for pancreatic lipase, in comparison with the positive control orlistat as the reference compound (IC_50_ of 2.149 ± 0.008).

### 3.5. Prediction of Pharmacokinetic and Toxicological Properties

**Figure 4:**
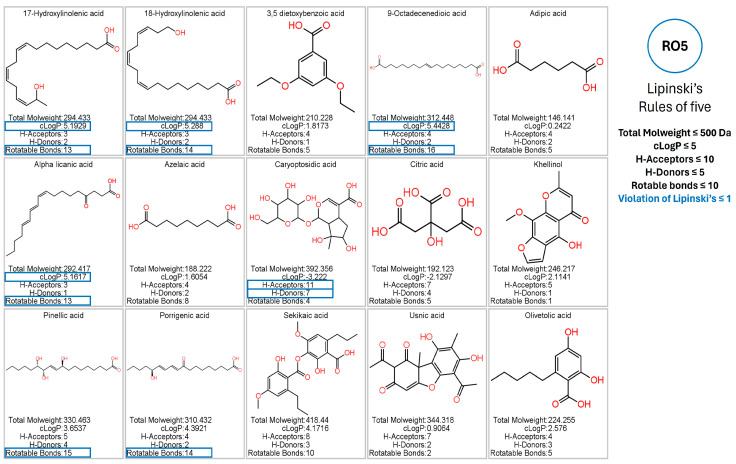
Evaluation of the pharmacokinetic properties based on Lipinski’s rules using the Osiris Data Warrior software of the phytochemicals identified in the *G. regalis* species.

Using the Osiris Data Warrior program, the in silico results of the pharmacokinetic and toxicological analysis of the phytochemicals identified from the *G. regalis* species were obtained. Phytochemicals that were potentially considered as inhibitors of the enzymes α-amylase, α-glucosidase, and human pancreatic lipase could be regarded as drugs administered orally; they should present at most one violation of Lipinski’s rule and will not present any risk of toxicity (mutagenicity, tumorigenicity, irritability, and reproductive effects) [40,41]. The results showed that of the 15 phytochemicals that were identified by UHPLC/MS, 5 were those that presented more than one violation in Lipinski’s rule (17-hydroxy linolenic acid, 18-hydroxy linolenic acid, 9-octadecenoic acid, alpha licanic acid, and caryoptosidic acid) (Figure 4).

**Figure 5:**
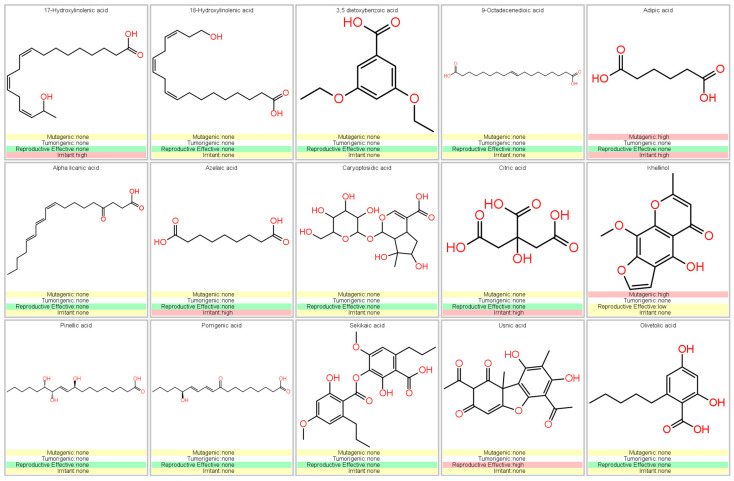
Analysis of toxicological risks (mutagenicity, tumorigenicity, reproductive effects, and irritant effects) using the Osiris Data Warrior software of the phytochemicals identified in the *G. regalis* species.

The compounds that did not present a maximum violation of Lipinski’s rule were 3,5-dietoxybenzoic acid, adipic acid, azelaic acid, citric acid, khellinol, pinellic acid, porrigenic acid, sekikaic acid, usnic acid, and olivetolic acid (Figure 4). However, the compound adipic acid presented a high risk of mutagenic and irritation due to the fragmentation that the carboxyl functional groups of the chemical structure can suffer (Figure 5). The compounds azelaic acid and citric acid presented a high risk of irritability; this, like the adipic acid compound, is because the carboxylic groups of its chemical structure can present fragmentation, thus generating highly irritating radicals (Figure 5). The khellinol compound showed a high risk of mutagenicity due to the fragment involving the benzopyranone rings and the oxidation of the methoxyl and hydroxyl groups. The compound that also presented a risk of toxicity was usnic acid; the fragment that involves the ketone group and the methyl group makes usnic acid present effects on reproduction (Figure 5).

Compounds that complied with Lipinski’s rules and that did not present any risk of toxicity (3,5-dietoxybenzoic acid, olivetolic acid, pinellic acid, porrigenic acid, and sekikaic acid) were considered as possible inhibitors of α-amylase, α-glucosidase, and human pancreatic lipase (Figure 5). Therefore, they were evaluated by in silico analysis and assessed their performance as inhibitors, comparing them with the reference inhibitors (acarbose for α-amylase and α-glucosidase; orlistat and MUP for human pancreatic lipase).

The prediction of pharmacokinetic and toxicological properties of sekikaic acid and related phytochemicals were evaluated to determine their potential as drug candidates. Assessment of absorption, distribution, metabolism, excretion, and toxicity (ADMET) parameters revealed that sekikaic acid exhibited favorable drug-like properties, supporting its potential therapeutic applications.

### 3.6. Docking Studies

#### 3.6.1. Molecular Docking of Phytochemicals for α-Amylase Inhibition

After the pharmacological and toxicological analysis, a molecular docking analysis was carried out to determine which compounds could be candidates as inhibitors of the enzymes α-amylase, α-glucosidase, and human pancreatic lipase.

**Figure 6:**
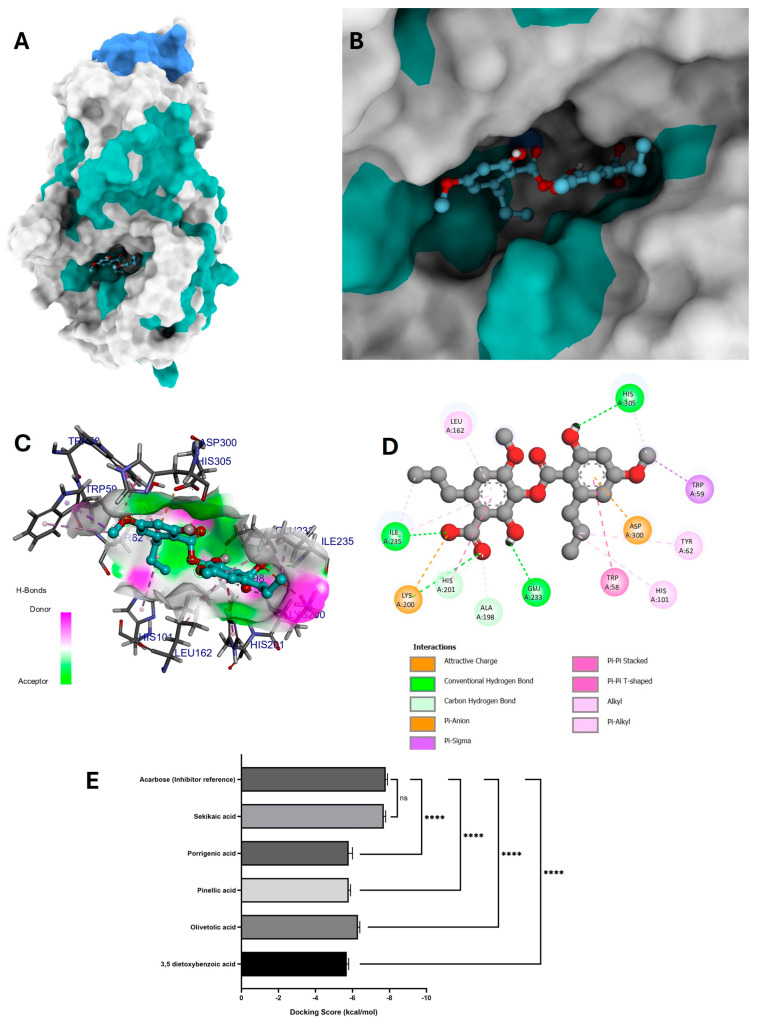
Molecular docking between the compound sekikaic acid and α-amylase. (**A**) Adopted molecular geometry of the sekikaic acid compound in the catalytic pocket of the α-amylase enzyme; (**B**) zoom view of the geometry adopted by the compound sekikaic acid in the catalytic pocket of α-amylase; (**C**) analysis of hydrogen bonds of the sekikaic acid–α-amylase complex; (**D**) map of predominant interactions of the molecular docking of the compound sekikaic acid and α-amylase; (**E**) binding energies of the identified compounds of the *G. regalis* species and the reference inhibitor acarbose. A one-way ANOVA was performed with a Dunnet test of multiple comparisons where the asterisks above the standard error of the mean bars between the groups indicate that the differences were statistically significant at *p* < 0.0001 (****) and ns = there is no significant difference.

For this analysis, three molecular docking runs were performed, obtaining the 10 most stable conformations and selecting the conformation with the highest binding affinity. Compounds that did not present any risk of toxicity and no more than one violation of Lipinski’s rules (3,5-dietoxybenzoic acid, olivetolic acid, pinellic acid, porrigenic acid, and sekikaic acid) were evaluated in a molecular docking analysis to observe their behavior as potential α-amylase inhibitors (Figure 6E).

All these compounds were compared with the reference inhibitor acarbose, which exhibited a binding affinity of −7.8 ± 0.1 kcal/mol. Among the tested compounds, sekikaic acid showed the most similar behavior to the reference inhibitor, with a binding affinity of −7.7 ± 0.1 kcal/mol.

The visual representation of the highest affinity conformation of sekikaic acid in the catalytic pocket of the α-amylase enzyme and its placement within the binding pocket are shown in Figure 6A,B. The interaction map of sekikaic acid in the catalytic pocket is shown in Figure 6D.

Sekikaic acid–amylase interactions exhibited four conventional hydrogen bonds: one between the amino acid Lys200 and the oxygen of the carbonyl group of the carboxylic part, allowing the acceptance of the hydrogen bond (Figure 6C,D). The hydroxyl group of sekikaic acid enabled the donation of a hydrogen bond between this functional group and the amino acid Glu233. The third hydrogen bond occurred between the amino acid Ile235 and the oxygen of the carboxylic group, while the fourth hydrogen bond was formed when the hydroxyl group of one of the aromatic rings donated its hydrogen to interact with the amino acid His305 (Figure 6C,D).

Additionally, sekikaic acid displayed an attractive charge interaction between the oxygen of the carboxylate group and the amino acid Lys200, as well as a π–anion interaction between the π electrons of the aromatic ring and the amino acid Asp300.

#### 3.6.2. Molecular Docking of Phytochemicals for α-Glucosidase Inhibition

**Figure 7:**
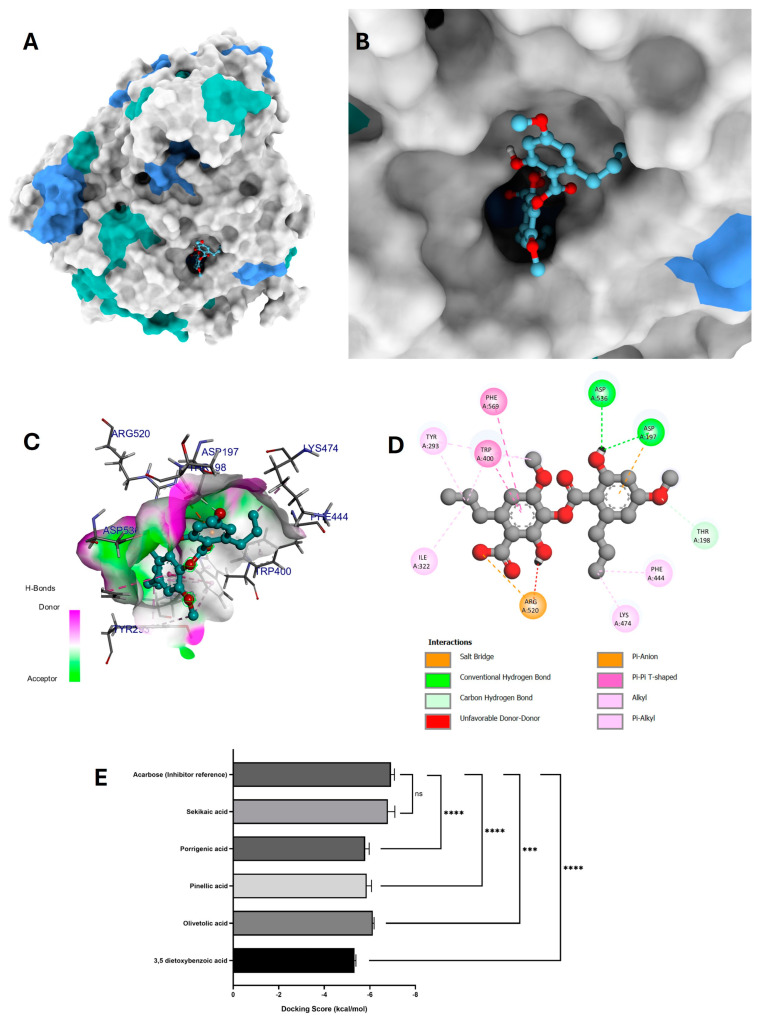
Molecular docking between the compound sekikaic acid and α-glucosidase. (**A**) Adopted molecular geometry of the sekikaic acid compound in the catalytic pocket of the α-glucosidase enzyme; (**B**) zoom view of the geometry adopted by the compound sekikaic acid in the catalytic pocket of α-glucosidase; (**C**) analysis of hydrogen bonds of the sekikaic acid–α-glucosidase complex; (**D**) map of predominant interactions of the molecular docking of the compound sekikaic acid and α-glucosidase; (**E**) binding energies of the identified compounds of the *G. regalis* species and the reference inhibitor acarbose. A one-way ANOVA was performed with a Dunnet test of multiple comparisons where the asterisks above the standard error of the mean bars between the groups indicate that the differences were statistically significant at *p* < 0.001 (***), or *p* < 0.0001 (****) and ns = there is no significant difference.

The results of the in silico analysis of *G. regalis* phytochemicals were obtained to determine which compounds could be candidate inhibitors of the α-glucosidase enzyme. For this analysis, the compound acarbose was used again as a reference inhibitor of the α-glucosidase enzyme (Figure 7E).

As in the molecular docking analysis of α-amylase, the compound that presented greater stability and binding affinity in the catalytic pocket of α-glucosidase was sekikaic acid (Figure 7E). This compound exhibited a favorable conformation in the catalytic pocket of α-glucosidase (Figure 7A,B) and showed good stability and affinity to the binding site (−6.8 ± 0.3 kcal/mol) due to the interactions it established within the catalytic pocket.

In the interaction map (Figure 7D), sekikaic acid exhibited two hydrogen bond interactions, both formed by donating hydrogen from the hydroxyl group of the aromatic ring to the amino acids Asp197 and Asp537 (Figure 7C,D). In addition to these interactions, sekikaic acid formed a salt bridge-type interaction between the oxygen of the carboxylate group and the amino acid Arg520, as well as a π–anion interaction between the π electrons of the aromatic ring and the amino acid Asp197 (Figure 7D).

Two T-shaped π–π interactions were observed between the π electrons of the aromatic ring of sekikaic acid and the amino acids Trp400 and Phe569. Six alkyl-type interactions were also identified: three occurred between the methylene group of the alkyl chain linked to the aromatic ring and the amino acids Tyr293, Ile322, and Trp400; another alkyl-type interaction was observed between the methyl group of methoxyl and the amino acid Trp400. The remaining two alkyl-type interactions involved the methyl group of one of the alkyl chains and the amino acids Phe444 and Lys474 (Figure 7D).

Furthermore, the interaction map revealed an unfavorable donor–donor interaction between the hydrogen of the hydroxyl group and the amino acid Arg520, as well as a carbon–hydrogen interaction between the hydrogen of the hydroxyl group and the amino acid Thr198. These interactions contributed to an adequate geometry and significant binding affinity in the catalytic site of α-glucosidase (Figure 7A,B).

The compounds that presented lower binding affinities compared to the reference inhibitor acarbose were 3,5-dietoxybenzoic acid (−5.3 ± 0.1 kcal/mol), olivetolic acid (−6.1 ± 0.1 kcal/mol), pinellic acid (−5.9 ± 0.2 kcal/mol), and porrigenic acid (−5.8 ± 0.2 kcal/mol) (Figure 7E).

#### 3.6.3. Molecular Docking of Phytochemicals for Lipase Pancreatic Inhibition

Molecular docking analysis was carried out between *G. regalis* phytochemicals (3,5-dietoxybenzoic acid, olivetolic acid, pinellic acid, porrigenic acid, and sekikaic acid) and the human pancreatic lipase enzyme. For the analysis, the reference inhibitors MUP and orlistat were used, as they are recognized inhibitors of human pancreatic lipase. These inhibitors were used to compare the *G. regalis* phytochemicals and assess their potential effectiveness in inhibiting human pancreatic lipase.

**Figure 8:**
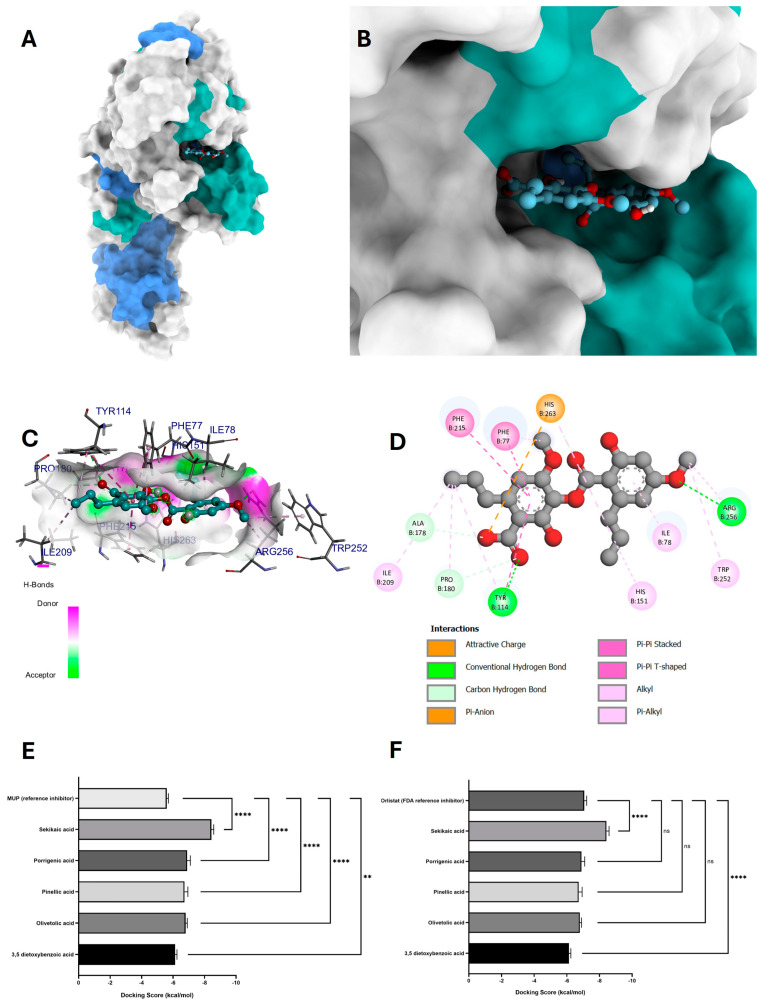
The molecular docking between the compound sekikaic acid and human pancreatic lipase. (**A**) Adopted molecular geometry of the sekikaic acid compound in the catalytic pocket of the human pancreatic lipase enzyme; (**B**) zoom view of the geometry adopted by the compound sekikaic acid in the catalytic pocket of human pancreatic lipase; (**C**) analysis of hydrogen bonds of the sekikaic acid human pancreatic lipase complex; (**D**) map of predominant interactions of the molecular docking of the compound sekikaic acid and human pancreatic lipase; (**E**,**F**) binding energies of the identified compounds of the *G. regalis* species and the references inhibitors MUP y orlistat. A one-way ANOVA was performed with a Dunnet test of multiple comparisons where the asterisks above the standard error of the mean bars between the groups indicate that the differences were statistically significant at *p* < 0.01 (**), or *p* < 0.0001 (****) and ns = there is no significant difference.

Figure 8E,F show the results of the binding affinities of the *G. regalis* phytochemicals and the reference inhibitors MUP and orlistat in the molecular docking analysis for the human pancreatic lipase enzyme. The molecular docking analysis revealed that the binding affinities of the tested compounds (3,5-dietoxybenzoic acid, olivetolic acid, pinellic acid, porrigenic acid, and sekikaic acid) were higher (−6.1 ± 0.1 kcal/mol, −6.8 ± 0.1 kcal/mol, −6.7 ± 0.2 kcal/mol, −6.9 ± 0.2 kcal/mol, and −8.4 ± 0.2 kcal/mol, respectively) compared to the reference inhibitors MUP and orlistat (−5.6 ± 0.1 kcal/mol and −7.1 ± 0.2 kcal/mol, respectively) (Figure 8E,F).

Among the tested compounds, sekikaic acid exhibited the highest binding affinity (−8.4 ± 0.2 kcal/mol) and the most stable conformation in the catalytic pocket (Figure 8A,B). Figure 8D shows the interaction map of sekikaic acid in the catalytic site of the enzyme, demonstrating two hydrogen bond interactions between the oxygen of the carbonyl group of the carboxylate with the amino acid Tyr114 and the oxygen of the hydroxyl group with the amino acid Arg256. These interactions allowed the acceptance of hydrogen bonds, as observed in Figure 8C.

Additionally, sekikaic acid exhibited an attractive charge interaction between the oxygen of the carboxylate group and the amino acid His263, which plays a direct role in human pancreatic lipase inhibition. The compound also formed eight alkyl-type interactions with the amino acids Phe77, His151, Tyr114, Pro180, Ile209, Trp252, Arg256, and His263, as well as one π-alkyl interaction between the π electrons of the aromatic ring and the amino acid Ile78.

Furthermore, two stacked π–π interactions were observed between the π electrons of the amino acids Phe77 and Phe215 with the π electrons of the aromatic ring of sekikaic acid, along with a π–π shaped interaction between the π electrons of the amino acid Tyr114 and the aromatic ring of sekikaic acid (Figure 8D).

### 3.7. Molecular Dynamic Simulation

**Figure 9:**
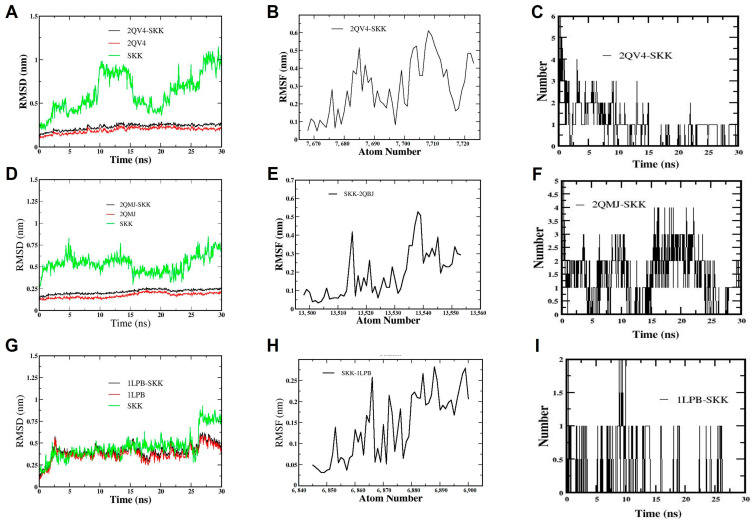
Results obtained from molecular dynamics simulation over a 30 ns simulation period of the compound sekikaic acid with the proteins α-amylase (PDB ID: 2QV4), α-glucosidase (PDB ID: 2QMJ), and human lipase pancreatic (PDB ID: 1LPB). (**A**–**C**) Root mean square deviation (RMSD), root mean square fluctuation (RMSF), and number of hydrogen bonds, respectively, of the sekikaic acid and α-amylase complex (PDB ID: 2QV4); (**D**–**F**) root mean square deviation (RMSD), root mean square fluctuation (RMSF), and number of hydrogen bonds, respectively, of the sekikaic acid and α-glucosidase complex (PDB ID: 2QMJ); (**G**–**I**) root mean square deviation (RMSD), root mean square fluctuation (RMSF), and number hydrogen bonds, respectively, of the sekikaic acid and human pancreatic lipase complee (PDB ID: 1LPB).

Molecular dynamics (MD) analyses were performed to evaluate the inhibitory behavior of sekikaic acid towards human pancreatic α-amylase, α-glucosidase, and human pancreatic lipase enzymes (Figure 9). To further assess the inhibitory capacity of sekikaic acid, which exhibited the most promising results in the molecular docking analyses (Figure 6, Figure 7 and Figure 8), MD simulations up to 30 ns were performed on each of the enzyme systems evaluated in the molecular docking (α-amylase, α-glucosidase, and human pancreatic lipase) (Figure 9).

#### 3.7.1. Molecular Dynamic Simulation of Sekikaic Acid for α-Amylase Inhibition

The conformation of the sekikaic acid compound and the α-amylase protein remained in a relatively stable state as the molecular dynamics simulation progressed (Figure 9A,B). It was also observed that the sekikaic acid compound, evaluated individually, presented fluctuations in the RMSD values in the range from 0.25 nm to 1.2 nm during molecular dynamics due to the rotational motion of the molecule. However, these RMSD fluctuations decreased (from 0.17 nm to 0.25 nm) when the sekikaic acid compound docked in the catalytic site of α-amylase, generating a significant conformational stability within the catalytic site of the protein (Figure 9A).

The RMSF of the sekikaic acid system and the α-amylase protein (2QV4) were evaluated during 30 ns of simulation (Figure 9B), showing that both reached equilibrium (from 0.05 nm to 0.6 nm) during the MD analysis, which aligns with the results obtained in the RMSD (Figure 9A).

#### 3.7.2. Molecular Dynamic Simulation of Sekikaic Acid for α-Glucosidase Inhibition

The results of the 30 ns MD simulation of the sekikaic acid compound and α-glucosidase (2QMJ) are shown in Figure 9D–F. Similar to the α-amylase results (Figure 9A–C), the RMSD values of the sekikaic acid and α-glucosidase complex showed significant stability (from 0.17 nm to 0.25 nm) throughout the simulation period (Figure 9D). The behavior of the sekikaic acid compound during this simulation exhibited a similar pattern to that observed in the α-amylase simulation (Figure 9A–C), with fluctuations in the RMSD values due to the torsional motion of the molecule (Figure 9D). However, when the sekikaic acid compound bound to the catalytic site of the α-glucosidase enzyme, it stabilized throughout the simulation due to the geometric conformation it adopted at this catalytic site and the interactions present at this binding (Figure 9D).

The RMSF values remained constant (from 0.1 to 0.5 nm) in the sekikaic acid and α-glucosidase complex during the simulation, indicating minimal fluctuations of the complex (Figure 9E).

#### 3.7.3. Molecular Dynamic Simulation of Sekikaic Acid for Human Pancreatic Lipase Inhibition

The sekikaic acid compound exhibited the highest stability in the catalytic site of human pancreatic lipase (1LPB), as shown in the molecular docking results (Figure 8). To evaluate the stability of this complex over time, an MD analysis was performed with a simulation time of 30 ns. The RMSD results of the sekikaic acid complex showed that the compound remained stable throughout the simulation, presenting slight fluctuations in the RMSD (from 0.17 to 0.5 nm) (Figure 9G).

The RMSF values reflected the stability of the sekikaic acid complex with human pancreatic lipase, with values ranging between 0.2 nm and 0.27 nm (Figure 9H). The stability observed within the catalytic site was influenced by the presence of hydrogen bonds, which helped maintain the adopted conformation of sekikaic acid throughout the simulation (Figure 9I). The number of hydrogen bonds in the sekikaic acid–human pancreatic lipase complex ranged from 1 to 2 (Figure 9I). Comparing the molecular docking and MD simulation results, it can be inferred that the hydrogen bonds formed by sekikaic acid involved the residues Tyr114 and Arg256 (Figure 8D).

## 4. Discussion

### 4.1. Chemical Analysis

The chemical composition of *G. regalis* was identified using an ethanolic extract based on previous studies demonstrating the efficiency of this extraction method for profiling Antarctic lichens and assessing their biological activities [40,41,45,46,47]. The extract yield (4%) was comparable to that of other Antarctic species, such as *Umbilicaria antarctica* (3%), *Placopsis contortuplicata* (6%), *Ochrolechia frigida* (9.6%) [45], and *Cladonia chlorophaea* (2%) [41]. The extract exhibited a gummy appearance, characteristic of the presence of fatty acids and waxes, which have been frequently reported in metabolomic studies of Antarctic lichens [40,41,45,46,47].

The compounds identified in *G. regalis* have previously been detected in metabolomic analyses of Antarctic lichens, including *Lecania brialmontii*, *Pseudephebe pubescens*, *Sphaerophorus globosus* [40], *Cladonia gracilis*, *C. chlorophaea*, *C. metacorallifera* [41,48], *Psoroma antarcticum*, *P. hypnorum* [46], *Umbilicaria antarctica*, *Ochrolechia frigida*, *Placopsis contortuplicata* [45], and *Himantormia lugubris* [47]. These species are characterized by the predominance of aromatic compounds, carbohydrates, lipids, depsides, depsidones, dibenzofurans, and chromones. The findings are based on advanced UHPLC/MS techniques, which have also been used to evaluate the antioxidant, anti-inflammatory, and enzymatic effects of these compounds [49]. Chemical profiling in lichens not only highlights the relevance of bioactive compounds for bioprospecting but also facilitates chemotaxonomic classification, the discovery of new therapeutic applications, and the optimization of extraction methods to obtain a greater diversity of chemical constituents [50,51].

The total phenolic content (TPC) of *G. regalis* differs from values reported for other Antarctic lichen genera such as *Lecania*, *Pseudephebe*, *Sphaerophorus*, *Cladonia*, *Psoroma*, *Umbilicaria*, *Ochrolechia*, *Placopsis*, *Himantormia*, and *Usnea* [40,41,45,46,47,52]. In these species, TPC ranges from as low as 0.279 ± 0.005 mg GAE/g to as high as 1000.6 ± 0.01 mg GAE/g in *L. brialmontii* and *O. frigida*, respectively. The concentration in *G. regalis* is comparable to that reported for *Cladonia gracilis* (53.563 ± 0.04 mg GAE/g), *Psoroma hypnorum* (46.174 ± 0.009 mg GAE/g), *Himantormia lugubris* (47.4 ± 0.05 mg GAE/g), and *Usnea aurantiaco-atra* (68.61 ± 0.01 mg GAE/g) [41,46,48,52].

Following the chemical profiling of the ethanolic extract of *G. regalis*, in vitro antioxidant and enzymatic inhibition assays were conducted to evaluate two key biological activities of Antarctic lichen extracts: their potential therapeutic applications for central nervous system diseases and metabolic syndrome. Additionally, complementary in silico studies, including molecular docking and molecular dynamics simulations, were performed to theoretically explore and predict the types of intermolecular interactions and the stability of bonds between the compounds in the extract and the active sites of the target enzymes.

### 4.2. Antioxidant Activity

In the DPPH assay, although *G. regalis* exhibited low efficacy based on its IC_50_ values, these results are consistent with those reported for extracts of *Evernia prunastri* (1926.3 ± 33.2 µg/mL), *Cladonia uncialis* (>2500 µg/mL), and *Parmelia sulcata* (669.3 ± 11.8 µg/mL), as well as for isolated compounds such as evernic, usnic, and salazinic acids, which have IC_50_ values exceeding 750 µg/mL [53]. However, comparisons with other lichen species, such as *Stereocaulon tomentosum*,* Lobaria pulmonaria*, *Cetraria islandica*, *Umbilicaria hirsuta*, *Xanthoria elegans*, and *Pseudevernia furfuracea* [54], as well as *Vulpicida pinastri* [55], indicate that antioxidant activity varies significantly depending on the solvent used for extraction (e.g., methanol, ethanol, acetone, dichloromethane, or hexane) [56,57,58].

### 4.3. Inhibitory Activity

The results for α-glucosidase are highly significant and comparable to those reported for the Antarctic species *Ochrolechia frigida* (16 ± 0.015 µg/mL) and *Psoroma hypnorum* (18.921 ± 0.005 µg/mL), as well as close to *Cladonia gracilis* (91.323 ± 0.010 µg/mL) [41,45,46], confirming the high antidiabetic potential of lichen extracts and compounds. Regarding α-amylase and pancreatic lipase, the values for *G. regalis* are moderately significant and contrast with those reported for *C. gracilis*, *C. chlorophaea*, *P. hypnorum*, *P. antarcticum*, *O. frigida*, *Umbilicaria antarctica*, and *Placopsis contortuplicata* [41,45,46].

The high inhibitory capacity of the *G. regalis* extract on α-glucosidase is also comparable to the activity exhibited by aromatic compounds isolated from *Parmotrema cristiferum*, with values ranging from 24.0 to 171 µg/mL [59]. Similarly, hexane extracts of *Evernia prunastri* showed comparable inhibition rates of 80.69% and 94.18% at concentrations of 3 mg/mL and 5 mg/mL, respectively [60]. The major presence of sekikaic acid in the extract may contribute significantly to its inhibitory action due to its strong antihyperglycemic activity [61]. Its potent effect is similar to the inhibition ranges observed in isolated compounds from species as *Hypotrachyna cirrhata* (from 30% to >80%) [62], *Roccella montagnei* (from 7.9 to >300 µM) [63], *Usnea baileyi* (from 10.4 to >200 µM) [64], *Ramalina conduplicans* (from 5% to >60%) [65], *Parmotrema tsavoense* (from 10.7 to 17.6 µM) [66], and *Parmotrema tinctorum* (from 74.7 to 98.2 µg/mL) [67].

Recent studies on Antarctic lichens have revealed a clear correlation between the inhibition potential mechanism of digestive enzymes, particularly α-glucosidase, total phenolic content, and antioxidant activity. This relationship has been observed in the ethanolic extracts of species such as *Cladonia gracilis* (IC_50_ inhibition = 91.323 ± 0.010 µg/mL; TPC = 55.563 ± 0.004 mg GAE/g; IC_50_ DPPH = 296.737 ± 0.021 µg/mL), *Ochrolechia frigida* (IC_50_ inhibition = 16 ± 0.015 µg/mL; TPC = 1000.6 ± 0.01 mg GAE/g; IC_50_ DPPH = 307.981 ± 0.053 µg/mL), and *Psoroma hypnorum* (IC_50_ inhibition = 18.921 ± 0.005 µg/mL; TPC = 46.174 ± 0.009 mg GAE/g; IC_50_ DPPH = 380.543 ± 0.011 µg/mL) [42,45,46]. These values are comparable to those obtained for the *G. regalis* extract (IC_50_ inhibition = 19.49 ± 0.027 µg/mL; TPC = 31.9 ± 0.016 mg GAE/g; IC_50_ DPPH = 2246.149 ± 0.086 µg/mL), which contains sekikaic acid as one of the major compounds in the extract and, according to the in silico evaluation, showed the best interaction and stability with the enzymes.

In previous studies [10], cytotoxic activity analysis of sekikaic acid has been performed; these results showed that sekikaic acid was inactive against A2780 ovarian and MCF-7 breast cancer cell lines [10]. In vitro studies recognize sekikaic acid as an inhibitor of α-glucosidase; in the intestine, this enzyme slows down digestion and the overall rate of glucose absorption in the blood [65]. However, further toxicological studies are required to confirm long-term safety and metabolic stability.

In addition, sekikaic acid meets Lipinski’s rule of five, indicating its potential oral bioavailability. The molecular weight of sekikaic acid is within the acceptable range (<500 Da), with a logP value suggesting good membrane permeability. Furthermore, the compound meets both hydrogen bond donor and acceptor criteria, reinforcing its drug-like potential [68]. These results align with previously published studies on natural inhibitors such as flavonoids and depsides, which also satisfy these pharmacokinetic criteria and exhibit strong enzyme inhibition properties [40].

Future research should focus on in vivo validation of the pharmacokinetic behavior of sekikaic acid, including metabolic stability, bioavailability, and potential adverse effects. Such studies will provide a more complete understanding of its therapeutic feasibility and pave the way for its application in drug development.

### 4.4. Molecular Docking

#### 4.4.1. Molecular Docking of Phytochemicals for α-Amylase Inhibition

Docking studies revealed that sekikaic acid exhibited a strong binding affinity for α-amylase, comparable to the reference inhibitor acarbose. Molecular interactions observed, including multiple hydrogen bonds and π–anion interactions, suggest that sekikaic acid can effectively stabilize within the catalytic pocket of the enzyme; this stability is crucial for its potential inhibitory activity [69].

Sekikaic acid demonstrated the most favorable interaction profile, reinforcing its potential as a natural inhibitor of α-amylase. The observed hydrogen bonding with key amino acids, particularly Lys200, Glu233, Ile235, and His305, suggests a strong and stable binding mode, which may contribute to its efficacy [70].

Furthermore, the presence of charge interactions, particularly between the oxygen of the carboxylate group and Lys200, and the π–anion interaction with Asp300, provides additional support for the high stability of sekikaic acid in the enzyme’s active site [45].

Additionally, docking studies have demonstrated that other polyphenolic com-pounds, such as flavonoids, can also inhibit α-amylase effectively due to their ability to form strong hydrogen bonds and hydrophobic interactions [71]. These results suggest that sekikaic acid could be a promising candidate for further studies in the development of α-amylase inhibitors derived from phytochemicals.

#### 4.4.2. Molecular Docking of Phytochemicals for α-Glucosidase Inhibition

Molecular docking analysis demonstrated that sekikaic acid had the highest binding affinity among the tested phytochemicals, making it the most promising inhibitor of α-glucosidase [72]. The observed interactions, including hydrogen bonding with Asp197 and Asp537, as well as π–anion and salt bridge interactions, contributed to its high stability in the enzyme’s catalytic pocket [47].

In comparison to acarbose, sekikaic acid exhibited a slightly lower but still significant binding affinity. The presence of π-π interactions with Trp400 and Phe569, along with multiple alkyl-type interactions, further supports its strong molecular interactions, which may enhance its inhibitory potential [41].

Although other phytochemicals such as 3,5-dietoxybenzoic acid, olivetolic acid, pinellic acid, and porrigenic acid also demonstrated some degree of binding affinity, their lower binding energies suggest they are less effective inhibitors of α-glucosidase compared to sekikaic acid [70]. The unfavorable donor–donor interaction with Arg520 and the carbon–hydrogen interaction with Thr198 observed for sekikaic acid highlight potential areas for molecular modifications to improve its binding efficiency [40].

Overall, these findings suggest that sekikaic acid is a promising candidate for further studies on natural α-glucosidase inhibitors, with potential applications in managing carbohydrate metabolism-related disorders [69].

The results of in silico studies on sekikaic acid could be compared with the available literature data regarding similar inhibitory effects of other depsides, such as lecanoric acid, identified by UHPLC/ESI/QToF/MS in *G. regalis*. Previous studies have suggested that lecanoric acid exhibits α-glucosidase and α-amylase inhibitory activity, although its binding affinity and enzyme interactions have not been extensively explored computationally [45,73].

Studies on other depsides, such as barbatic acid and divaricatic acid, have indicated that their hydroxyl and carboxyl functional groups contribute to their ability to form hydrogen bonds and hydrophobic interactions, which are key for enzyme inhibition [74,75]. Comparing their binding energies and interaction profiles with sekikaic acid would provide further insight into its potential superiority as an inhibitor.

#### 4.4.3. Molecular Docking of Phytochemicals for Human Pancreatic Lipase Inhibition

The molecular docking results indicate that sekikaic acid has the highest potential as a human pancreatic lipase inhibitor among the tested *G. regalis* phytochemicals [45]. Its strong binding affinity (−8.4 ± 0.2 kcal/mol) exceeded that of orlistat (−7.1 ± 0.2 kcal/mol), a widely used lipase inhibitor [47]. The ability of sekikaic acid to interact with key catalytic amino acids (Phe215, Arg256, and His263) suggests a stable and effective inhibition mechanism [70].

The hydrogen bonding interactions with Tyr114 and Arg256, along with an attractive charge interaction with His263, likely contribute to the strong enzyme inhibition [40]. Moreover, the π–π stacking and π–alkyl interactions observed between sekikaic acid and multiple amino acids further enhance its conformational stability within the catalytic site [41].

In comparison to the other phytochemicals analyzed, sekikaic acid demonstrated superior molecular interactions, suggesting it as the most promising candidate for natural inhibition of human pancreatic lipase [69]. The molecular docking analysis supports the hypothesis that sekikaic acid could serve as an effective alternative to synthetic inhibitors, making it a potential lead compound for further investigation in lipase inhibition research [71].

The computational analysis identified 3,5-dietoxybenzoic acid, olivetolic acid, pinellic acid, porrigenic acid, and sekikaic acid as potential inhibitors of α-amylase, α-glucosidase, and human pancreatic lipase, based on Lipinski’s rule of five [40,41]. Among these, sekikaic acid exhibited the highest binding affinity, suggesting strong inhibitory potential [40,76].

However, a comparison with experimental data is necessary to confirm its effectiveness. Previous studies on flavonoids and depsides have shown significant α-glucosidase inhibition, yet no direct validation exists for sekikaic acid [65]. Similarly, research on phenolic acids suggests a correlation between docking scores and enzyme inhibition, but sekikaic acid’s superiority over acarbose and orlistat remains unverified [77].

While docking provides valuable insights, experimental validation is crucial to confirm sekikaic acid’s efficacy and compare it with existing natural inhibitors [41]. Future studies should integrate in vitro assays to strengthen these findings.

### 4.5. Molecular Dynamics (MD) Simulation

The molecular interaction and dynamic behavior of sekikaic acid at the catalytic site of the different enzyme systems (α-amylase, α-glucosidase, and human pancreatic lipase) were evaluated by the root mean square deviation (RMSD), root mean square fluctuation (RMSF), and hydrogen bonds (HBonds) parameters (Figure 9). These parameters allowed us to gain knowledge about the conformational and energetic stability of sekikaic acid in each of the enzymatic systems evaluated (α-amylase, α-glucosidase, and human pancreatic lipase) (Figure 9). With the molecular dynamic simulation of sekikaic acid for α-amylase inhibition, in addition to the evaluation of the RMSD and RMSF parameters, the number of hydrogen bond interactions was determined throughout the simulation and compared with the results obtained in the molecular docking. The sekikaic acid compound presented from one to four hydrogen bonds during the 30 ns simulation (Figure 9C). Comparing these results with the molecular docking (Figure 6D), the hydrogen interactions occurred with the amino acids Lys200, Glu233, Ile235, and His305, which confer stability to sekikaic acid within the catalytic site (Figure 6B,D and Figure 9C). These results indicate that sekikaic acid acquires a stable conformation during MD, allowing it to bind effectively to the catalytic site of α-amylase (2QV4), highlighting its potential as a possible α-amylase inhibitor candidate.

The number of hydrogen bonds formed in the sekikaic acid and α-glucosidase complex (Figure 9F) ranged from one to four hydrogen bonds. However, during most of the simulation time, the number of hydrogen bonds formed was between one and two (Figure 9F), which, according to the results shown in the molecular docking (Figure 7D), correspond to the hydrogen bonds formed by the residues Asp197 and Asp536. These residues are directly involved in the inhibition of α-glucosidase. These results indicate that the sekikaic acid compound demonstrates stability and favorable interactions within the catalytic site, supporting its potential as an α-glucosidase inhibitor candidate.

The behavior exhibited by sekikaic acid suggests that it can be considered a candidate inhibitor of the human pancreatic lipase enzyme. The MD simulations provided crucial insights into the interactions and stability of sekikaic acid within the evaluated enzymes (α-amylase, α-glucosidase, and human pancreatic lipase), supporting its potential as a natural inhibitor with antidiabetic effects. However, further studies are suggested to complement these therapeutic effects and fully elucidate the pharmacological profile of sekikaic acid.

According to the molecular docking results for the inhibition of α-amylase, α-glucosidase, and human pancreatic lipase enzymes, sekikaic acid demonstrated the highest binding affinities among the analyzed compounds (Figure 6, Figure 7 and Figure 8). This compound exhibited significant interactions at the catalytic sites of these enzymes, highlighting its potential as a leading candidate for antidiabetic therapies and further investigations into its therapeutic properties. Similar findings have been reported in lichen metabolites, such as those from *Placopsis contortuplicata*, *Ochrolechia frigida*, and *Umbilicaria antarctica*, which have demonstrated enzymatic inhibition and antioxidant activity through molecular docking techniques and in vitro methods [78,79].

In all docking analyses, sekikaic acid showed higher affinities compared to reference inhibitors such as acarbose, MUP, and orlistat, indicating a strong interaction between the enzymes and this compound. The molecular docking analysis of human pancreatic lipase revealed that most of the compounds identified in the *G. regalis* species exhibited superior inhibitory behavior compared to the reference inhibitors, as shown in Figure 8E,F. This suggests that these compounds, especially sekikaic acid, have the potential to effectively inhibit human pancreatic lipase and generate a therapeutic effect. Similar results have been observed in studies of *Ficus lutea* and *Piper betle*, where molecular docking demonstrated strong binding affinities of plant-derived compounds with enzymatic targets related to diabetes [79,80,81].

Molecular dynamics simulations validated the stability of sekikaic acid within the catalytic pockets of α-amylase, α-glucosidase, and human pancreatic lipase over a 30 ns simulation period. The conformational and energetic stability of sekikaic acid was confirmed, aligning with research on natural inhibitors like usnic acid and depsides, where molecular docking and dynamics simulations have demonstrated stable and effective binding to enzymatic targets [81,82]. Toxicological and pharmacokinetic analyses indicated that sekikaic acid complies with Lipinski’s rules and presents no significant risks, further supporting its viability as a drug-like candidate. This is consistent with previous studies on other lichen-derived metabolites, such as those from *Cladonia* species, which have demonstrated low toxicity profiles alongside strong bioactivity [78,81,83]. Furthermore, it aligns with pharmacokinetic evaluations of plant-based inhibitors, such as those from *Cinnamomum zeylanicum*, which have shown similar stability and safety profiles when applied to diabetes-related targets [84,85].

## 5. Conclusions

This study represents the first report on the chemical profile of the ethanolic extract of the Antarctic lichen *Gondwania regalis*, as well as its antioxidant and antidiabetic potential. Characterization by UHPLC/ESI/QToF/MS, allowed the identification of 21 compounds belonging to chemical groups characteristic of Antarctic lichens. In vitro assays revealed moderate antioxidant activity and significant inhibition of α-glucosidase, highlighting the potential of this species for developing natural therapies against oxidative stress and diabetes.

Among the identified compounds, sekikaic acid emerged as the most promising candidate, exhibiting strong interactions with biological targets based on molecular docking and molecular dynamics analyses. Furthermore, toxicological and pharmacokinetic evaluations provided insights into its safety and therapeutic viability.

Overall, these findings enhance knowledge of secondary metabolites in Antarctic lichen species and validate their bioactivity, laying the foundation for future studies. Future studies should focus on evaluating the biological effects of major compounds in cellular and animal models, alongside pharmacodynamic investigations to elucidate their mechanisms of action. Additionally, the development of biotechnological production and chemical synthesis strategies could enhance their pharmaceutical applications.

## Figures and Tables

**Table 1:** Identification by UHPLC/ESI/QToF/MS of the metabolites present in the ethanolic extract of *G. regalis*.

Peak	Retention Time (min)	Tentative Identification	[M-H]^−^	Theoretical Mass (*m*/*z*)	Measured Mass (*m*/*z*)	Accuracy (ppm)	Metabolite Type	MS Ions (ppm)
1	1.34	Mannitol	C_6_H_13_O_6_	181.0712	181.0723	6.07	C	151.0598
2	3.21	Citric acid	C_6_H_7_O_7_	191.0192	191.0184	−4.19	OA	111.0074
3	14.46	Azelaic acid	C_9_H_15_O_4_	187.0775	187.0769	−3.21	FA	-
4	14.72	Unknown	C_25_H_27_O	179.0311	179.0321	5.58	-	165.0923
5	16.33	Khellinol	C_13_H_9_O_5_	245.0489	245.0431	−23.67	C	165.0914
6	17.56	9-Octadecenedioic acid	C_18_H_31_O_4_	311.2227	311.2228	0.32	FA	-
7	18.23	Pinellic acid	C_18_H_33_O_5_	329.2333	329.2345	3.64	FA	251.0674, 215.1239
8	19.54	Pinellic acid isomer	C_18_H_33_O_5_	329.2333	329.2345	3.64	FA	251.0674, 215.1239
9	20.22	2,4-Dihydroxy-6-pentylbenzoate(Olivetolic acid)	C_12_H_15_O_4_	223.0983	223.0981	−0.89	FA	165.0923
10	21.01	Unknown	C_26_H_16_O_4_	392.1075	392.1054	−5.36	-	350.0945
11	22.03	Lecanoric acid *	C_16_H_13_O_7_	317.0666	317.0653	−4.10	d	167.034
12	22.50	3,5-Dietoxybenzoic acid	C_11_H_13_O_4_	209.0822	209.0823	0.48	A	163.0360
13	22.77	Caryoptosidic acid	C_16_H_23_O_11_	391.1231	391.1245	3.58	I	311.2178, 263.1603
14	23.78	Adipic acid	C_16_H_27_O_4_	283.1914	283.1869	−15.89	OA	273.1797
15	24.37	Sekikaic acid	C_22_H_25_O_8_	417.1553	417.1571	4.31	d	247.16944
16	25.19	17-Hydroxylinolenic acid	C_18_H_29_O_3_	293.2122	293.2136	4.77	FA	243.19740
17	25.31	Porrigenic acid	C_18_H_29_O_4_	309.2070	309.2091	6.79	FA	291.19653
18	26.13	Usnic acid *	C_18_H_15_O_7_	343.0823	343.0822	−0.29	DBF	295.2291; 231.0647; 328.0570
19	26.93	18-Hydroxylinoleic acid	C_18_H_31_O_3_	295.2278	295.2279	0.34	FA	277.2133
20	27.7	18-Hydroxylinolenic acid	C_18_H_29_O_3_	293.2122	293.2071	−17.39	FA	243.19740
21	31.2	Alpha licanic acid	C_18_H_27_O_3_	291.1965	291.1907	−19.92	FA	265.1444

* Identified by co-spiking experiments using authentic standard compounds. C = carbohydrates; OA = organic acid; FA = fatty acid; d = depside; A = aromatic; I = iridoid; DBF = dibenzofuran.

**Table 2:** Total phenolic content (TPC) and antioxidant activity of the extract of lichen *G. regalis*.

Assay	TPC (mg GAE/g)	FRAP (µmol Trolox/g)	ORAC (µmol Trolox/g)	DPPH IC_50_ (µg/mL)
*G. regalis*	31.9 ± 0.016 *	6.802 ± 0.062 *	13.463 ± 0.15 *	2246.149 ± 0.086 *
Gallic acid ^#^	-	-	-	2.24 ± 0.04

Values marked with * are statistically different (*p* < 0.05). ^#^ Positive control.

**Table 3:** Enzyme inhibitory activity of the extract of lichen *G. regalis*.

Assay	α-Glucosidase IC_50_ (µg/mL)	α-Amylase IC_50_ (µg/mL)	Pancreatic Lipase IC_50_ (µg/mL)
*G. regalis*	19.49 ± 0.027 *	585.216 ± 0.026 *	326.451 ± 0.066 *
Orlistat ^#^	-	-	2.149 ± 0.008 *
Acarbose ^#^	206.614 ± 0.008 *	6.477 ± 0.003 *	-

Values marked with * are statistically different (*p* < 0.05). ^#^ Positive control.

## Data Availability

The datasets presented in this study can be found in online repositories. The name of the repository and accession number can be found at: MetaboLights—MTBLS10281.
